# DNA Dumbbell and Chameleon Silver Nanoclusters for miRNA Logic Operations

**DOI:** 10.34133/2020/1091605

**Published:** 2020-03-02

**Authors:** Yiting Jiang, Peng Miao

**Affiliations:** ^1^Suzhou Institute of Biomedical Engineering and Technology, Chinese Academy of Sciences, Suzhou 215163, China; ^2^University of Science and Technology of China, Hefei 230026, China

## Abstract

Multiplex miRNA analysis is a fundamental issue for exploring a complex biological system and early diagnosis of miRNA-related diseases. Herein, we have developed a series of novel logic gates for miRNA analysis coupling DNA nanostructures and chameleon silver nanoclusters (AgNCs). DNA dumbbell structures are firstly designed with two independent nucleation sequences for AgNCs at the 5′ and 3′ ends, respectively. By introducing different miRNA inputs, separations of two AgNCs are controlled and the fluorescence property of AgNCs changes. By studying the ratiometric fluorescence responses, sensitive and selective analysis of multiple miRNAs can be achieved. The present work provides powerful tools for miRNA diagnostics and may also guide future DNA nanostructure-based logic gates.

## 1. Introduction

miRNAs are noncoding endogenous RNA molecules with a length of 18~25 nucleotides, which are partially or completely complementary to the 3′ or 5′ untranslated region (UTR) of target genes [1, 2]. miRNAs play a vital role in a wide range of biological processes including cell proliferation, differentiation, and apoptosis [3–7]. In addition, increasing evidences have been revealed demonstrating that the occurrences of many diseases including cancers are closely associated with abnormal expression of specific miRNAs [8], e.g., prostate cancer-associated miR-21 and miR-141 [9], breast cancer-associated miR-155-3p [10], and liver fibrosis-associated miR-378 [11]. Therefore, miRNAs are considered to be promising biomarker candidates in the early diagnosis and prognosis of various cancers [12–14]. Quantitative detection of abnormal miRNA expressions in cells, tissues, urine, or blood may have significant applications for clinical diagnosis [15]. However, the inherent properties of miRNA including its short length, low abundance, and high sequence homology increase the difficulty of accurate and reliable analysis, which place great demands on modern analytical tools. Moreover, since multiple miRNAs are involved in the regulatory networks, single target detection cannot accurately reflect the occurrence of biological processes. The development of simple, sensitive, and selective approaches for multiplex analysis of miRNAs is in urgent need [16].

Due to the nanoscale precision and full addressability of DNA structures [17], DNA logic gates have been developed, which could implement traditional integrated circuit functions at the molecular scale and reflect the information of multiple inputs of molecules. So far, a number of DNA logic-gate-based biosensors have been constructed [18]. By careful designs of DNA structures, unique output can be obtained in responding to multiple miRNA inputs after certain logic operations. For example, Li et al. applied a DNA nanotweezer to build logic gates [19]; Liu et al. integrated G-quadruplex/hemin DNAzyme as the output of logic operations [20]; and Peng et al. fabricated DNA prism-based logic gates for specific recognition and computing on target cell surfaces [21]. On the other hand, metal nanoclusters have attracted considerable attention due to their unique physical, electrical, and optical properties [22]. In particular, DNA-templated silver nanoclusters (AgNCs) are excellent signal output candidates for DNA logic gates, which show the merits of facile synthesis, high-luminescence quantum yield, tunable fluorescence emission, and good photostability [23–25]. In a previous report, we have also demonstrated that AgNCs possess excellent biocompatibility like carbon nanodots, which are suitable for applying in biological systems [26–28].

The emphasis of this work is the construction of DNA dumbbell structures containing two nucleation sequences of chameleon AgNCs for miRNA logic operations. An enzyme-free and label-free process is involved after the recognition of target miRNAs, which improves the stability and practical utility of this system. In addition, although several independent logic gates are developed based on different DNA structures, the same signal threshold of output is shared, which provides possibilities for the combination of these logic gates as a series of analytical tools for miRNA diagnostics.

## 2. Results

**Figure 1:**
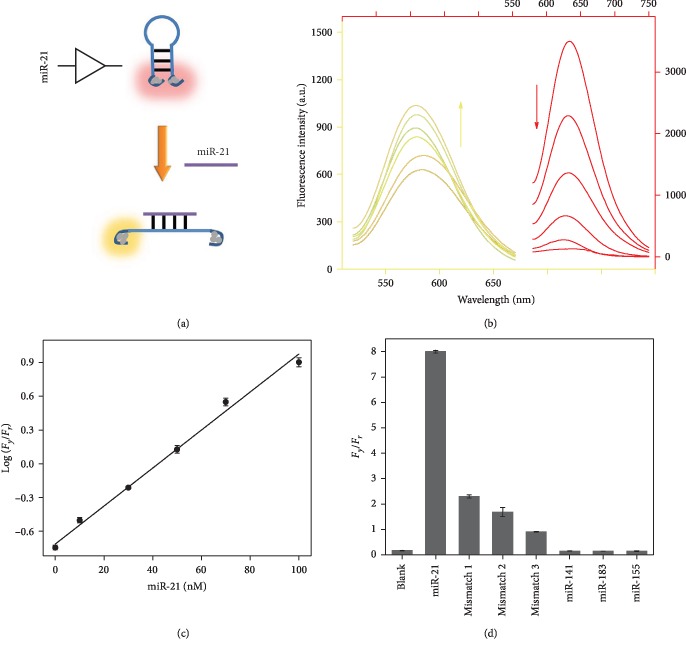
(a) Schematic illustration of the AgNC-based miR-21 assay. (b) Fluorescence emission spectra of probe Y-templated AgNCs for the detection of miR-21 (0, 10, 30, 50, 70, and 100 nM). (c) Calibration plot reflecting the relationship between the logarithmic ratio of fluorescence peaks and miR-21 concentration. (d) Selectivity investigation of the miR-21 assay.

miR-21 is firstly selected as a proof-of-concept target miRNA in a AgNC-based fluorescence assay (Figure 1(a)). A hairpin-structured DNA probe (probe Y) is designed with two nucleation sequences for AgNCs, in which one emits a strong yellow fluorescence and the other shows no fluorescence. The TEM image demonstrates the successful synthesis of the DNA-templated nanoclusters (Figure S1). With the two types of AgNCs approaching each other, the yellow fluorescence decreases while a new red emission rises [29]. Since the loop sequence of probe Y is complementary to miR-21, after a specific hybridization reaction, the hairpin structure is opened and the two AgNCs are separated. As a result, the yellow fluorescence is enhanced while the red fluorescence intensity decreases, which are highly correlated with the concentration of miR-21 (Figure 1(b)). By studying the ratio of the fluorescence peaks (*F*_*y*_ and *F*_*r*_), a linear relationship is established between logarithmic *F*_*y*_/*F*_*r*_ and miR-21 concentration (Figure 1(c)). The limit of detection is calculated to be 5.4 nM, which is quite excellent without any signal amplification. To further evaluate the selectivity of this ratiometric fluorescent assay, interfering miRNAs (miR-141, miR-183, and miR-155) and some mismatched sequences are examined under the same experimental conditions. The results verify that the fluorescence color change from red to yellow can only be induced by target miR-21. The other miRNAs fail to generate the chameleon phenomenon, suggesting that the strategy has potential to distinguish target miRNA from possibly interfering miRNAs (Figure 1(d)).

To obtain the best optical performances of the fluorescent logic systems, the experimental conditions are optimized on the following DNA templates including probe DOR, TOR, and TAND5, respectively. Different excitation wavelengths and DNA template concentrations are applied for the synthesis reactions. By comparing the fluorescence peaks, the optimized excitation wavelengths for probe DOR, TOR, and TAND5 are 560 nm, 540 nm, and 560 nm. The optimized concentrations of probe DOR, TOR, and TAND5 are 3 *μ*M, 3 *μ*M, and 1 *μ*M (Figures S2-S4).

**Figure 2:**
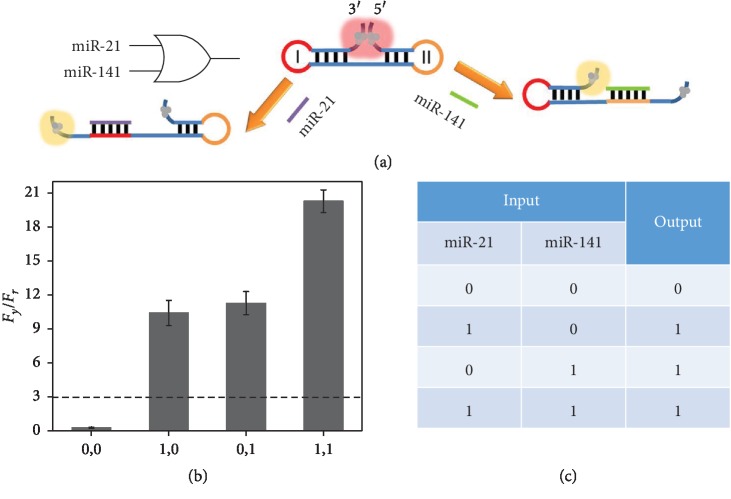
(a) Schematic illustration of the double-input OR gate. (b) The ratios of fluorescence peaks (*F*_*y*_/*F*_*r*_) of probe DOR-templated AgNCs in various input modes (*F*_*y*_ is the peak intensity at 560 nm, and *F*_*r*_ is the peak intensity at 612 nm). (c) Truth table of the double-input OR gate.

Numerous evidences have been explored, showing that multiple miRNAs are differentially expressed in certain cancers [30]. Therefore, we herein design a double-input OR gate for two types of miRNAs (miR-21, miR-141) in order to determine whether the sample contains any of these sequences.  Figure 2(a) illustrates the operating principle. Generally, the dumbbell-shaped DNA (probe DOR) is used as the template for the synthesis of AgNCs. With two hairpin structured regions, AgNCs at the 5′ and 3′ ends are in close proximity to produce a red fluorescence. The two loops (I and II) are designed to be the complementary sequences against miR-21 and miR-14, respectively. In the presence of miR-21, loop I hybridizes with the target miRNA and opens the hairpin structure. Similarly, with miR-141, a similar hybridization reaction occurs and the corresponding hairpin structure can also be opened. Therefore, in both cases, AgNCs at the 5′ and 3′ ends can be separated and the yellow emission is recovered. We have recorded the fluorescence responses in different input situations. The parameters of *F*_*y*_/*F*_*r*_ are calculated and compared in Figure 2(b). With the value larger than the threshold of 3, the output is defined as “1” or true, otherwise it is “0” or false. The truth table is summarized in Figure 2(c), which is logically correct, demonstrating the successful construction of the double-input OR gate.

**Figure 3:**
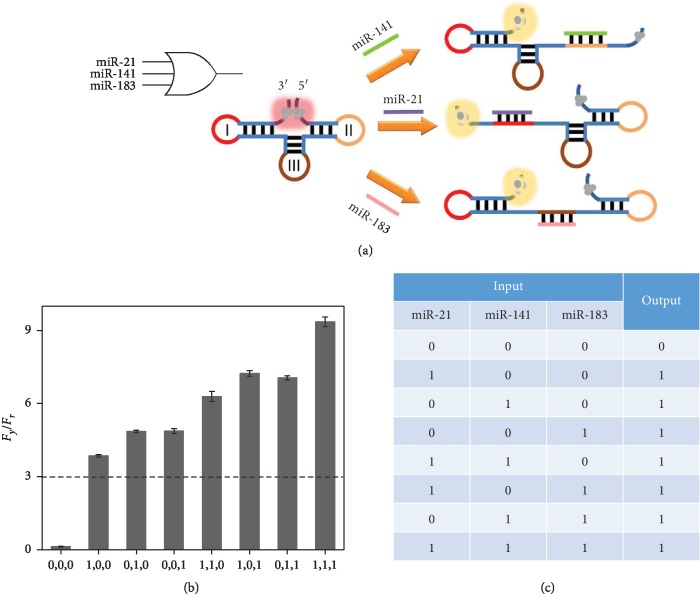
(a) Schematic illustration of the triple-input OR gate. (b) The ratios of fluorescence peaks (*F*_*y*_/*F*_*r*_) of probe TOR-templated AgNCs in various input modes (*F*_*y*_ is the peak intensity at 560 nm, and *F*_*r*_ is the peak intensity at 604 nm). (c) Truth table of the triple-input OR gate.

We have further designed DNA templates for triple-input logic operations. Three miRNAs, namely, miR-21, miR-141, and miR-183, are introduced as three examples of targets. As shown in Figure 3(a), a variant dumbbell-structured template (probe TOR) modified from probe DOR is designed, which contains three hairpin structures. The two AgNCs localized at the 5′ and 3′ ends are localized with close proximity as always. The three loops (I, II, and III) are complementary to the three target miRNAs, respectively. In this initial state, the red emission is much higher than that of the yellow emission, and the *F*_*y*_/*F*_*r*_ is below the threshold of 3. In the presence of any combination types of miR-21, miR-141, and miR-183, corresponding hairpin structures can be opened and the two AgNCs can no longer approach each other, which leads to the chameleon phenomenon and high *F*_*y*_/*F*_*r*_ ratio outputs (Figure 3(b)). The truth table is correct (Figure 3(c)), and this triple-input OR gate increases the diversity of miRNA detection types, which may help the improvement of the diagnosis accuracy.

**Figure 4:**
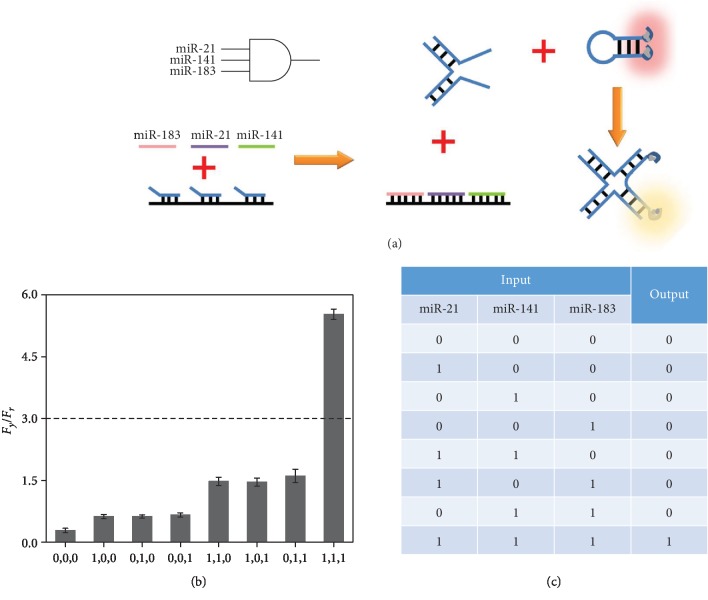
(a) Schematic illustration of the triple-input AND gate. (b) The ratios of fluorescence peaks (*F*_*y*_/*F*_*r*_) of probe TAND5-templated AgNCs in various input modes (*F*_*y*_ is the peak intensity at 560 nm, and *F*_*r*_ is the peak intensity at 615 nm). (c) Truth table of the triple-input AND gate.

In order to determine the coexistence of target miRNAs, a triple-input AND gate is further developed based on toehold-mediated strand displacements and four-way junction formation, which is an important supplementary for a miRNA assay (Figure 4(a)). Probes TAND1, TAND2, and TAND3 partially hybridize with probe TAND4, forming a double-stranded DNA complex. In addition, a hairpin-structured probe TAND5 is designed like probe Y with two nucleation sequences for AgNCs, which emit a red fluorescence. In the double-stranded DNA complex, probes TAND1, TAND2, and TAND3 can be displaced by target miR-21, miR-141, and miR-183 with completely complementary sequences, respectively. The three released strands can form a DNA four-way junction coupled with probe TAND5, which simultaneously separates two AgNCs at the 5′ and 3′ ends of TAND5. A chameleon phenomenon is thus observed, which reflects the coexistence of the three targets. The fluorescence responses in different input situations are studied. In the absence of any miRNA, the *F*_*y*_/*F*_*r*_ value cannot reach the threshold of 3 and the output of “0” is achieved. After the reactions with the combination of the three miRNAs, a much larger *F*_*y*_/*F*_*r*_ is generated (Figure 4(b)). The truth table of the AND gate is logically correct, which can be used for simultaneous detection of three target miRNAs (Figure 4(c)).

**Figure 5:**
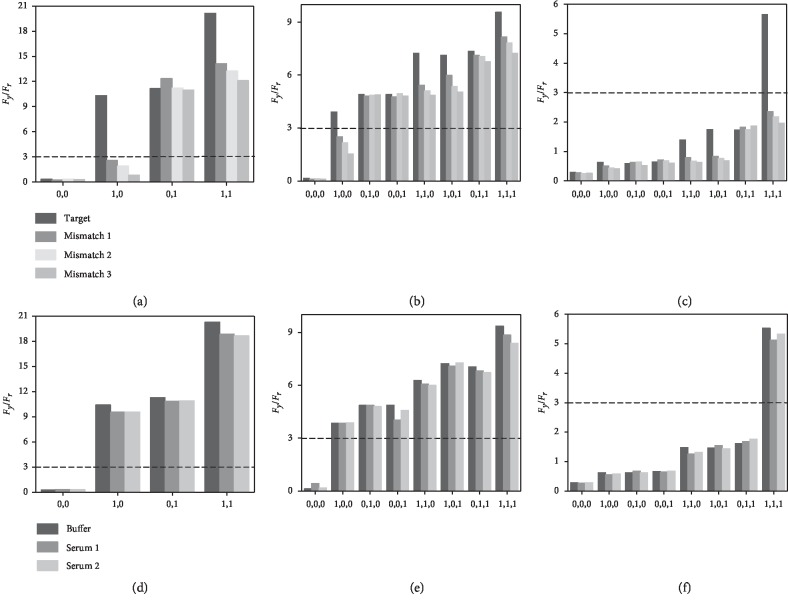
Selectivity investigation of the miRNA logic operations: (a) double-input OR gate, (b) triple-input OR gate, and (c) triple-input AND gate. Practical confirmation of the miRNA logic operations: (d) double-input OR gate, (e) triple-input OR gate, and (f) triple-input AND gate.

To verify the selectivity of the developed logic gates, we have employed three mismatched miRNAs to replace target miR-21 in the logic gates under the same experimental conditions. The *F*_*y*_/*F*_*r*_ responses are then measured and compared with target cases. For a double-input OR gate, lower *F*_*y*_/*F*_*r*_ values are observed for the input situations of (1,0) and (1,1). The irrational outputs of “0” are achieved for (1,0) since no target miR-21 or miR-141 is available to trigger the chameleon. In addition, although the outputs of “1” remain for (1,1), the decreased *F*_*y*_/*F*_*r*_ values indicate only miR-141 functions and the mismatched miRNAs cannot hybridize with the loop (Figure 5(a)). Similarly, for a triple-input OR gate, low *F*_*y*_/*F*_*r*_ values are observed for the input situations of (1,0,0), (1,1,0), (1,0,1), and (1,1,1), demonstrating the disabilities of the mismatched miRNAs in the logic OR operation (Figure 5(b)). As to the triple-input AND gate, since mismatched miRNAs cannot replace probe TAND2, no four-way junction structures can be formed and the conformation of probe TAND5 remains unchanged, which are demonstrated by the outputs of “0” (Figure 5(c)). All these results are good evidences for the high selectivity of the logic gates.

Most current logic devices may suffer drawbacks of weak anti-interference ability, which need to be operated under ideal experimental conditions. To test the practical utility of the proposed logic gates in complicated biological environments, they have been challenged with diluted human serum samples. Fluorescence responses are measured, and *F*_*y*_/*F*_*r*_ values are calculated. As shown in Figures 5(d)to 5(f), not only are the truth values in good accordance with corresponding values in ideal buffer conditions but the detailed *F*_*y*_/*F*_*r*_ values are also quite close to the standard values, which verify that the serum samples have limited effects on the results of the logic operations. Therefore, the developed logic gates may have great prospects for practical applications of multiple analysis of target miRNAs.

## 3. Discussion

We have successfully constructed a series of miRNA logic gates by combining chameleon AgNCs and DNA nanostructures, which show a number of merits. First, AgNCs with excellent biocompatibility are facilely in situ synthesized on DNA templates, which eliminate fluorescence labeling and the costs are significantly reduced. Second, the unique chameleon fluorescence property initiated by target-induced DNA structure transformations is subtly integrated in the sensing system. Third, the logic operations ensure simultaneous analysis of multiple miRNAs with the same threshold; meanwhile, high selectivity and good practical performance are achieved, which may have great potential utility for biochemical researches. We believe that the proposed strategy may offer a powerful analytical tool for miRNA analysis and encourages more in-depth applications of DNA nanotechnology.

## 4. Materials and Methods

### 4.1. Materials and Chemicals

Silver nitrate (AgNO_3_), sodium borohydride (NaBH_4_), and diethypyrocarbonate (DEPC) were purchased from Sigma-Aldrich (USA). Serum samples were collected from the local hospital. Other reagents were of analytical grade and used without further purification. All oligonucleotides were synthesized and purified by Sangon Biotechnology Co., Ltd. (Shanghai, China). The sequences are listed in Table S1. The oligonucleotides were dissolved in phosphate buffer solution (0.2 M, Na_2_HPO_4_/NaH_2_PO_4_, pH 7.4) to obtain stock solutions with the concentrations of 100 *μ*M. Double-distilled water was used throughout the experiments, which was firstly purified with a Millipore system under 18 MΩ cm resistivity and then treated with DEPC (0.1%). Fluorescence spectra were acquired with an F-7000 Fluorescence Spectrophotometer (Hitachi, Japan). The slit widths of the excitation and emission were both 10 nm.

### 4.2. Synthesis of DNA-Templated AgNCs

The nucleation sequences (probe Y, probe DOR, probe TOR, and probe TAND5) for AgNCs were heated at 95°C for 5 min and gradually cooled to room temperature. Then, these DNA templates were mixed with AgNO_3_ in phosphate buffer solution (pH 7.4), followed by vigorous shaking for 30 s. The mixtures were kept in the dark at 4°C for 60 min. Subsequently, freshly prepared NaBH_4_ was added to the above solutions with vigorous shaking for 60 s. The molar ratio of probe Y/AND5 : Ag^+^ : NaBH_4_ was 1 : 6 : 6. The molar ratio of probe DOR/TOR : Ag(NO)_3_ : NaBH_4_ was 1 : 8 : 8. Finally, the solutions were incubated in the dark at 4°C for 3 h before fluorescence measurements.

### 4.3. Optimization of Experimental Conditions

The excitation and emission wavelengths of all DNA-templated AgNCs were measured, and the optimized values were determined. DNA templates with different concentrations (probe DOR/TOR: 0.3, 0.5, 0.7, 1, 3, and 5 *μ*M; probe TAND5: 0.3, 0.5, 0.7, 1, 1.5, and 2 *μ*M) were used for the synthesis of AgNCs under the fixed ratios mentioned above. Then, fluorescence spectra were recorded and compared.

### 4.4. Logic Operations

Four logic gates were constructed including the YES gate, the double-input OR gate, the triple-input OR gate, and the triple-input AND gate. Target miRNAs (miR-21, miR-141, and miR-183) were selected as the inputs and chameleon AgNCs were used as the signal outputs. According to the logic operations, miRNA inputs with various combinations were mixed with DNA/AgNC complexes and the fluorescence spectra were recorded after incubating for 2 h at room temperature.

## Data Availability

All data is available in the main text or in the Supplementary Materials.

## Supplementary Materials

Supplementary Materials Figure S1: TEM image of DNA-templated AgNCs. Figure S2: optimization of the synthesis conditions of probe DOR-templated AgNCs: (a) excitation wavelength; (b) probe DOR concentration. Figure S3: optimization of the synthesis conditions of probe TOR-templated AgNCs: (a) excitation wavelength; (b) probe TOR concentration. Figure S4: optimization of the synthesis conditions of probe TAND5-templated AgNCs: (a) excitation wavelength; (b) probe TAND5 concentration. Table S1: DNA and RNA sequences used in this work.
