# Supplementary material for: DNA Dumbbell and Chameleon Silver Nanoclusters for miRNA Logic Operations
Source: Research (Wash D C). 2020 Mar 2;2020:1091605. doi: 10.34133/2020/1091605 (PMC7071348; doi:10.34133/2020/1091605)
Supplement: Supplementary Materials — Figure S1: TEM image of DNA-templated AgNCs. Figure S2: optimization of the synthesis conditions of probe DOR-templated AgNCs: (a) excitation wavelength; (b) probe DOR concentration. Figure S3: optimization of the synthesis conditions of probe TOR-templated AgNCs: (a) excitation wavelength; (b) probe TOR concentration. Figure S4: optimization of the synthesis conditions of probe TAND5-templated AgNCs: (a) excitation wavelength; (b) probe TAND5 concentration. Table S1: DNA and RNA sequences used in this work. [file 1091605.f1.docx]

**Supplementary Materials**

**DNA Dumbbell and Chameleon Silver Nanoclusters for miRNA Logic Operations**

Yiting Jiang^1,2^, and Peng Miao^1,2^

^1^ Suzhou Institute of Biomedical Engineering and Technology, Chinese Academy of Sciences, Suzhou 215163, China

^2^ University of Science and Technology of China, Hefei 230026, China

Correspondence should be addressed to Peng Miao; miaopeng@sibet.ac.cn


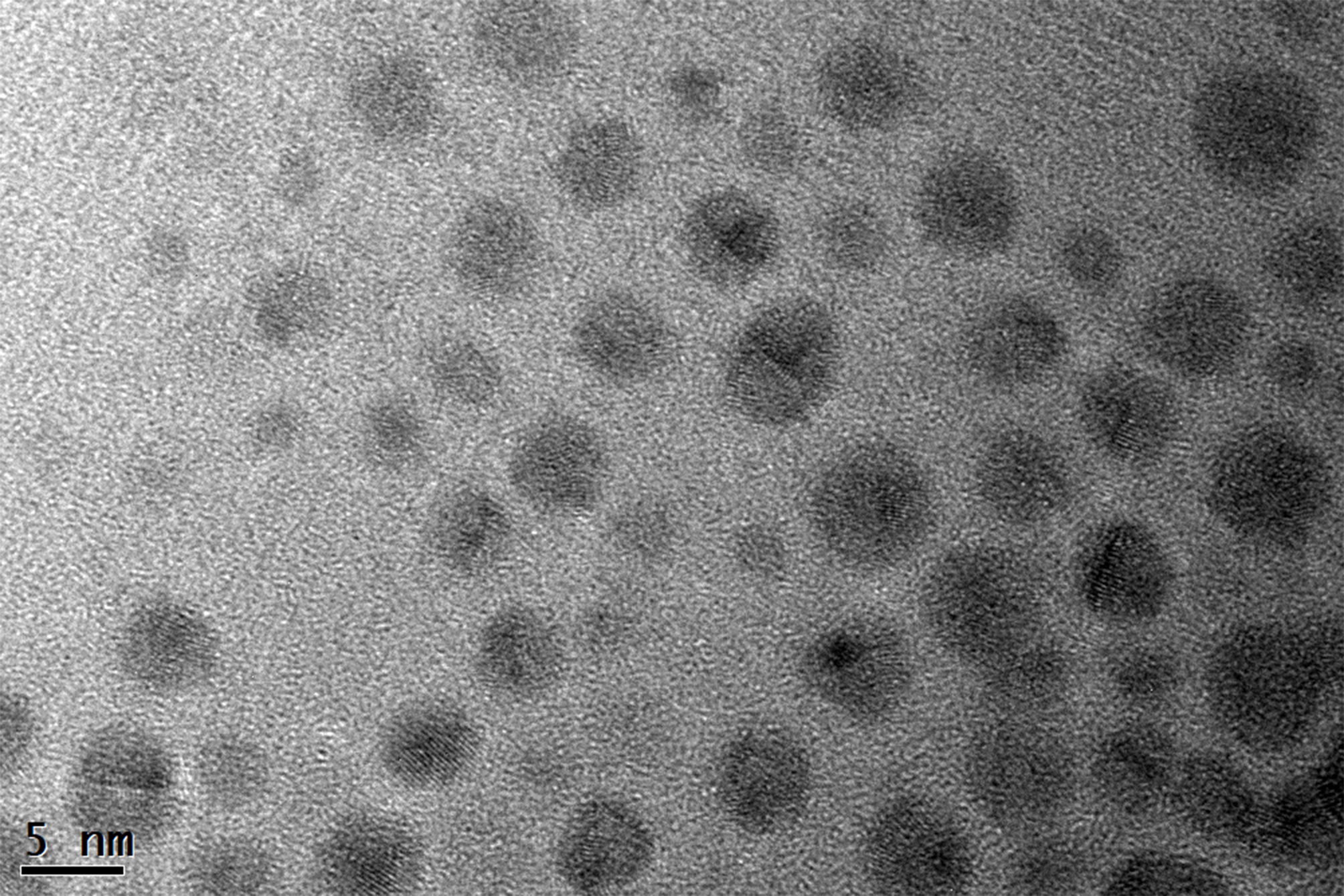


FIGURE S1: TEM image of DNA-templated AgNCs.


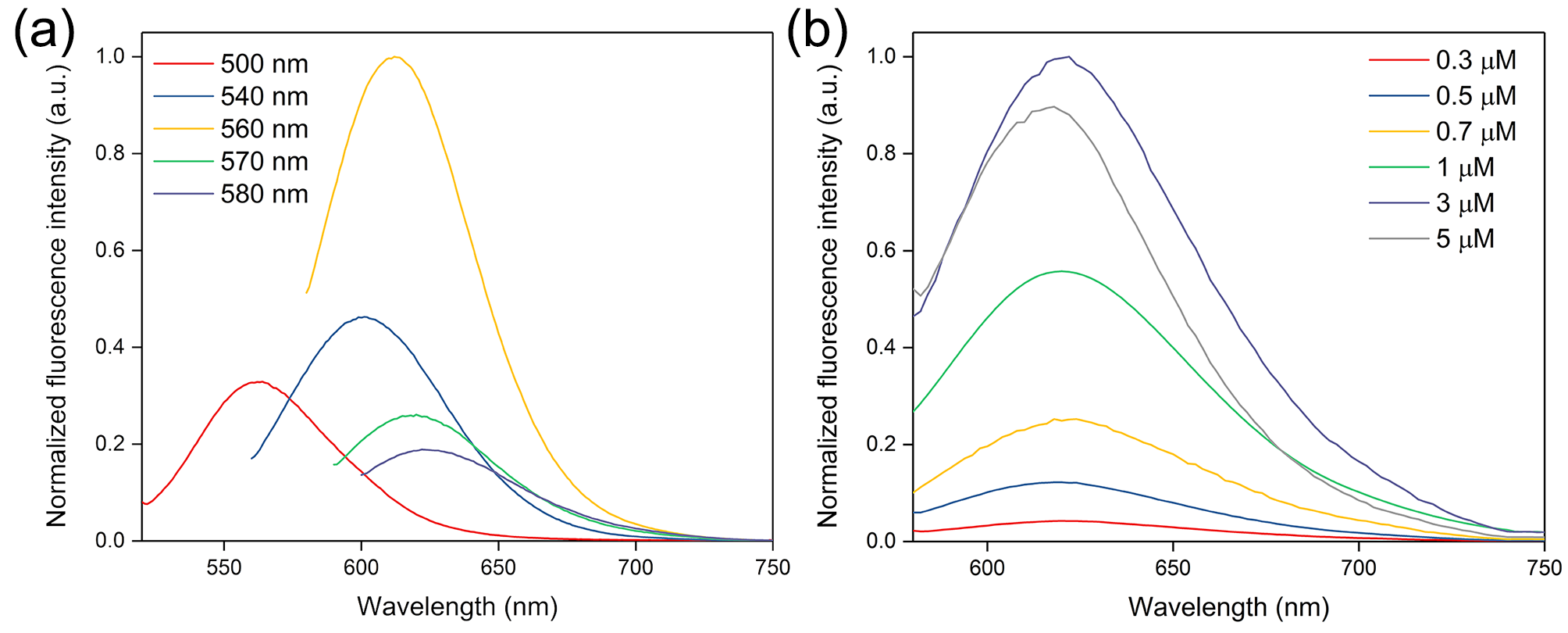


FIGURE S2: Optimization of the synthesis conditions of probe DOR-templated AgNCs: (a) excitation wavelength; (b) probe DOR concentration.


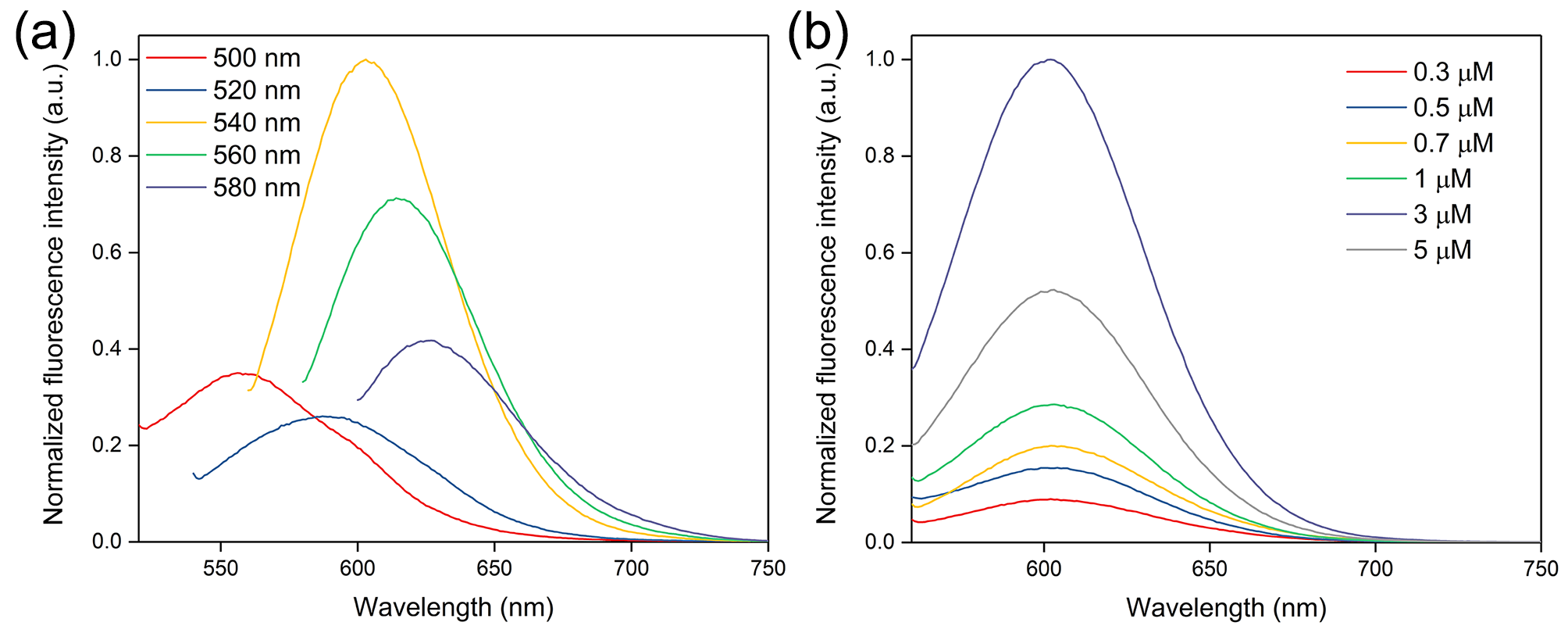


FIGURE S3: Optimization of the synthesis conditions of probe TOR-templated AgNCs: (a) excitation wavelength; (b) probe TOR concentration.


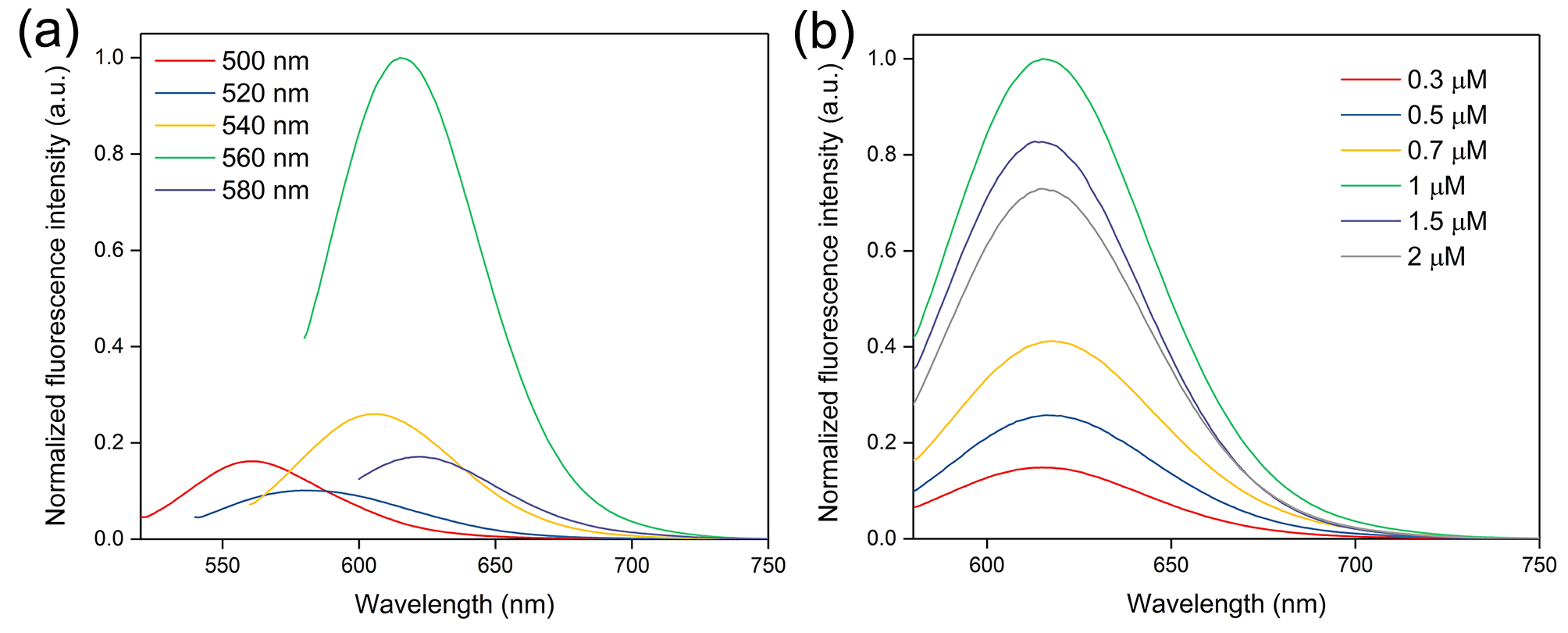


FIGURE S4: Optimization of the synthesis conditions of probe TAND5-templated AgNCs: (a) excitation wavelength; (b) probe TAND5 concentration.

TABLE S1: DNA and RNA sequences used in this work.

| Name | Sequence (from 5’ to 3’) |
| --- | --- |
| miR-21 | UAGCUUAUCAGACUGAUGUUGA |
| mismatch 1 | UAGCUUAUCAGACUGAAGUUGA |
| mismatch 2 | UAGCUUAUCAGACUGAACUUGA |
| mismatch 3 | UAGCUUAUCAGACUGAACGUGA |
| miR-141 | UAACACUGUCUGGUAAAGAUGG |
| miR-183 | UAUGGCACUGGUAGAAUUCACU |
| miR-155 | UUAAUGCUAAUCGUGAUAGGGGUU |
| probe Y | CCCCCTTAATCCCCCTTTTCTTCAACATCAGTCTGATAAGCTAAGAAAACCCCCTAATTCCCCC |
| probe DOR | CCCCCTTAATCCCCCTTTTCTAGGCCATCTTTACCAGACAGTGTTACCTAGAAAATATTTTTAGTCTCAACATCAGTCTGATAAGCTAGACTAAAAACCCCCTAATTCCCCC |
| probe TOR | CCCCCTTAATCCCCCTTTTCTACCATCTTTACCAGACAGTGTTATAGAAAATGGGGAGTGAATTCTACCAGTGCCATACCCCGTTTTTAGTCAACATCAGTCTGATAAGCTACTAAAAACCCCCTAATTCCCCC |
| probe TAND1 | **TAAAGATGG**AAACGCATCAG |
| probe TAND2 | **CTGATGTTG**TGAGAGAAAA |
| probe TAND3 | **GAATTCACT**GCTTTCCA |
| probe TAND4 | **AGTGAATTC**TACCAGTGCCATAT**CAACATCAG**TCTGATAAGCTA**CCATCTTTA**CCAGACAGTGTTA |
| probe TAND5 | CCCCCTTAATCCCCCTTTTCTCTCAAGTGCAGTGAATTCAGAAAACCCCCTAATTCCCCC |
